# Parental care contributes to vertical transmission of microbes in a skin-feeding and direct-developing caecilian

**DOI:** 10.1186/s42523-023-00243-x

**Published:** 2023-05-15

**Authors:** Marcel T. Kouete, Molly C. Bletz, Brandon C. LaBumbard, Douglas C. Woodhams, David C. Blackburn

**Affiliations:** 1 School of Natural Resources and Environment, University of Florida, Gainesville, FL 32611 USA; 2 Department of Natural History, Florida Museum of Natural History, University of Florida, Gainesville, FL 32611 USA; 3 Department of Biology, University of Massachusetts, Boston, MA 02125 USA

**Keywords:** 16S rRNA, Amphibia, Dermatophagy, Horizontal transmission, Skin and gut microbiomes, Vertical transmission

## Abstract

**Background:**

Our current understanding of vertebrate skin and gut microbiomes, and their vertical transmission, remains incomplete as major lineages and varied forms of parental care remain unexplored. The diverse and elaborate forms of parental care exhibited by amphibians constitute an ideal system to study microbe transmission, yet investigations of vertical transmission among frogs and salamanders have been inconclusive. In this study, we assess bacteria transmission in *Herpele squalostoma,* an oviparous direct-developing caecilian in which females obligately attend juveniles that feed on their mother’s skin (dermatophagy).

**Results:**

We used 16S rRNA amplicon-sequencing of the skin and gut of wild caught *H. squalostoma* individuals (males, females, including those attending juveniles) as well as environmental samples. Sourcetracker analyses revealed that juveniles obtain an important portion of their skin and gut bacteria communities from their mother. The contribution of a mother’s skin to the skin and gut of her respective juveniles was much larger than that of any other bacteria source. In contrast to males and females not attending juveniles, only the skins of juveniles and their mothers were colonized by bacteria taxa Verrucomicrobiaceae, Nocardioidaceae, and Erysipelotrichaceae. In addition to providing indirect evidence for microbiome transmission linked to parental care among amphibians, our study also points to noticeable differences between the skin and gut communities of *H. squalostoma* and that of many frogs and salamanders, which warrants further investigation.

**Conclusion:**

Our study is the first to find strong support for vertical bacteria transmission attributed to parental care in a direct-developing amphibian species. This suggests that obligate parental care may promote microbiome transmission in caecilians.

**Supplementary Information:**

The online version contains supplementary material available at 10.1186/s42523-023-00243-x.

## Background

Most of our knowledge of the skin and gut microbiomes of vertebrates—and their transmission between parents and offspring—is based on studies of mammals. Recent studies of the microbiomes of fishes and amphibians have advanced our understanding of the factors that affect microbiome colonization and microbial diversity among individuals and between species. In mammals, host phylogeny and diets are major factors driving differences in gut colonization [1, 2], as are traits such as powered flight [3]. Studies of mice have also reported sex-related differences in the gut microbiome linked to hormonal differences between males and females [4, 5]. Similarly, reproductive status, life stage, sex [6, 7], and their interaction [8, 9], and of sex and stable isotopes—a measure that can inform the trophic position of animal communities and often shows a 2–3% increase in ẟ^15^N per trophic level [10, 11]—can be important factors explaining microbiome differences in fishes. In amphibians, both environmental and life history factors can influence host microbiomes, though not uniformly. In salamanders and frogs, the environment clearly shapes the skin microbiome in some species [12, 13], but not in others [14, 15]. Life history, in contrast, is consistently found to shape amphibian skin and gut microbiomes [14, 16–18], as it does in other vertebrate taxa, especially those species with parental care [19–21]. Whereas the roles of the environment and life history have been explored, the extent to which parental care influences microbial colonization and diversity is less well known in amphibians.

Parental care often involves some form of food provisioning. In mammals, this typically includes milk, but across vertebrates might include regurgitated food, scales, skin fragments, and feces. Each of these contributes to vertical transmission of the microbiome between conspecifics and contrasts with the horizontal transmission established through interactions with the environment [22]. Among mammals, the most well-studied species is, of course, humans. Components of the human microbiome can be transmitted vertically between mother and infant, including through breast milk [23, 24], the mother’s vagina, stools, and oral cavity [25], or her skin [26–28]. Vertical microbiome transmission can also result from contact between infants and toys, pets, and caregivers [29, 30]. The bacterial genera *Bifidobacterium*, *Lactobacillus*, and *Staphylococcus* are abundant and shared between breastmilk and the infant gut, whereas *Lactobacillus* and *Prevotella* dominate the shared mother-infant skin microbial community. The infant gut microbiome is dynamic immediately following birth [31, 32], undergoing at least three stages of remodeling. These stages begin with a decrease, followed by stabilization, and then an increase in bacterial diversity and richness, ceasing at about nine months of age when the infant gut community matures and mostly resembles that of adults. However, early life colonization and bacterial dynamics in humans can be confounded by the birth mode (vaginal or C-section) or mother-inherited conditions (antibiotics medication, Inflammatory Bowel Disease, or Type II diabetes) which indicates that maternal health can impact infant microbiome colonization.

Because parental care varies among vertebrates, the well-studied patterns of microbiome transmission observed in humans are likely not directly applicable to other species. For example, female koalas (*Phascolarctos cinereus*) feed juveniles with a special form of feces called pap that has a different consistency and texture from normal adult feces [33]. Similarly, feces consumption (coprophagy) is documented in the Japanese rock ptarmigan (*Lagopus muta japonica*) and likely contributes to transgenerational microbial transfer [34]. In contrast to most other vertebrates that exhibit parental care, egg attendance in amphibians such as the four-toed salamander *Hemidactylium scutatum* [12] and the glass frog *Hyalinobatrachium colymbiphyllum* [17, 19] have not been found to clearly lead to transmission of the skin microbiome between parent and offspring. This suggests that though parental care in amphibians provides, for example, protection from predators or dehydration [35] and leads to transmitting innate defenses [36], it may not confer the same benefits to offspring with bacterial colonization that is observed in mammals. Yet, amphibians exhibit a diversity of unique parental care strategies and these likely vary in their impact on microbial transmission. Among amphibians, the remarkable forms of parental investment of caecilians remain unexplored for their potential role in vertical microbiome transmission.

**Fig. 1:**
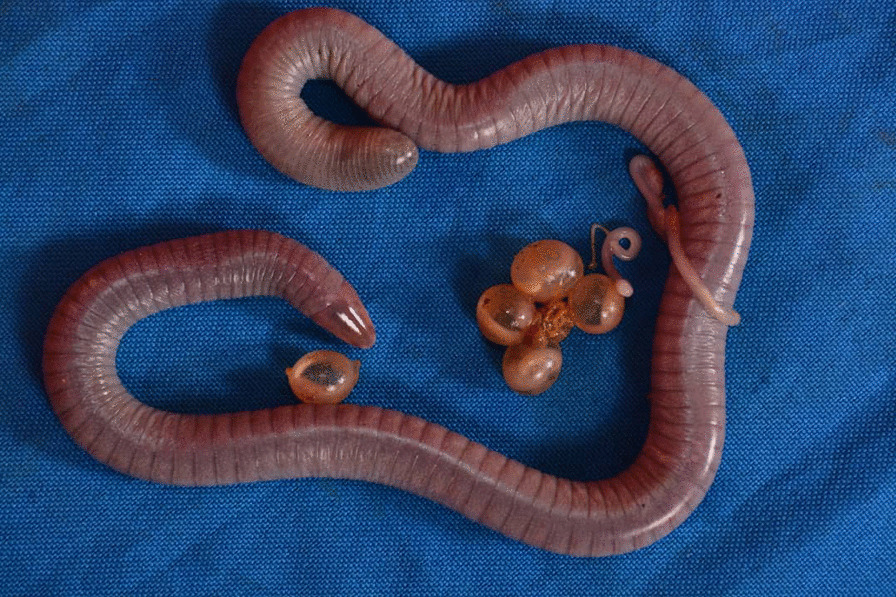
Adult *H. squalostoma* attending to a clutch of four eggs and three unpigmented juveniles

Caecilians (order Gymnophiona) are the least studied order of all living Amphibia, the vertebrate class that also includes frogs (Anura) and salamanders (Caudata). Caecilian life history strategies are remarkably distinct from other amphibians. At least some oviparous and direct-developing species—that lay eggs in soil or under leaves from which hatch miniature versions of the terrestrial adults—engage in parental care that includes eggs attendance followed by two distinct modes of provisioning offspring. In skin-feeding (dermatophagy), juveniles use their teeth to peel and then feed on fragments of the lipid-rich skin of their attending mother [37, 38]. In fluid-provisioning, the mother exudes a liquid from her cloacae that is consumed by her offspring [39, 40]. Only female caecilians have been observed to attend and provision juveniles and these interactions can last up to three months; well-documented examples include *Boulengerula taitanus* [38], *Herpele squalostoma* [41] (Fig. 1), *Microcaecilia dermatophaga* [37], and *Siphonops annulatus* [39]*.* While attending their offspring, there is an increase in lipid metabolization in the outermost layer of the mother’s epidermis (stratocorneum) and an enlargement of the epidermal cells leading to a pale coloration in the skin [38]. These changes are synchronized with sex hormones concentrations that play an important role in the process of skin remodeling [42]. Both parental care and physiological changes in females are well documented in several species, but it remains unknown whether and how these traits relate to the microbiomes of caecilians and the colonization processes in juveniles. The unique life history of caecilians provides an interesting opportunity for exploring source-sink patterns [43], including contributions to the juvenile’s microbiome from the environment and through maternal care.

In this study, we characterize the skin and gut microbiomes of *Herpele squalostoma* (family Herpelidae), a terrestrial direct-developing caecilian species from Central Africa that is oviparous and exhibits both maternal egg attendance and dermatophagy [41]. Because *H. squalostoma* engages in parental care involving egg attendance and skin-feeding, and potentially fluid-provisioning, this species provides an opportunity to investigate the role of parental care in vertical microbiome transmission in caecilians. We hypothesized that previously observed differences in diet between male and female *H. squalostoma* effect their skin and gut microbiomes and translate into a significant association between relative abundances in the microbiomes and ẟ^15^N. Also, if juvenile *H. squalostoma* feed on the skin of their mother, we expect their ẟ^15^N to be enriched relative to adults in general and females in specific. We assessed the extent to which maternal skin and gut microbiomes contribute to the microbial communities of the juvenile skin and gut. We also explored differences between adult males and females and the potential role of diet in explaining these differences. We expected similarities due to vertical microbiome transmission between the skin of the mother and gut of their juveniles (for skin-feeding) as well as between the mother’s gut and both their juvenile’s skin and gut (for fluid-provisioning). Based on comparisons to other vertebrates, we also expected that the skin and gut microbiomes of juveniles would be less diverse than those of adults. To investigate the role of the environment as a source for the skin microbiome, we assessed the relationships between the bacterial communities of the surrounding soil and the skin of both juvenile and adult *H. squalostoma*. We expected to find similarities between the bacteria in the environment and the skin. If so, this would reflect the ecology of *H. squalostoma*, which is known to be active below ground in tropical soils of Central Africa [44].

## Results

These results are based on a total of 29 individuals of *H. squalostoma* comprising 14 juveniles and 15 adults (nine females, six males). The nine females in our dataset include three attending females that were found guarding two, five, and six juveniles, respectively.

Sequences preprocessing with *QIIME* recovered a total of 1,582,454 sequences (range: 5686–51,769; mean = 23,976.6 ± 9700.7; mean frequency per feature = 299.7) and an additional 5,686 sequences from environmental samples. ANOVA (Additional file 1: Fig. S1) did not detect significant differences across life stage in the number of sequences of the skin (F = 3.43, df = 23, *p* = 0.08) or gut (F = 0, df = 16, *p* = 0.99) samples in our dataset.

### Characterization of microbiome communities of *Herpele squalostoma*

Our core bacteria analysis determined that two phyla dominated the skin of adult *H. squalostoma* (Table 1): Actinobacteria and Proteobacteria. In total, seven bacteria families dominated the skin community of adults *H. squalostoma*: Acetobacteracea, Brevibacteriaceae, Brucellaceae, Comamonadaceae, Hyphomicrobiaceae, Microbacteriaceae, and Nocardiaceae. The most dominant families were Comamonadaceae and Microbacteriaceae and the most abundant genus was *Comamonas* (Comamonadaceae). Two bacterial genera that are often reported to dominate the skin of other amphibians (i.e., frogs [15] and salamanders [13, 45]) were not present in our sample: *Acinetobacteria* (Moraxellaceae) and *Pseudomonas* (Pseudomonadaceae).

**Table 1:** Core bacteria of adults *H. squalostoma* skin and gut communities

Core bacteria of adults skin and gut communities	*p* value	Benjamini–Hochberg corrected *p* value
*Amplicon sequence variants of adults skin*		
p__Proteobacteria;c__Alphaproteobacteria;o__Rhizobiales;f__Brucellaceae	0.00055	0.041
p__Proteobacteria;c__Betaproteobacteria;o__Burkholderiales;f__Comamonadaceae;g__Comamonas	7.90E−05	0.01
p__Actinobacteria;c__Actinobacteria;o__Actinomycetales;f__Brevibacteriaceae;g__Brevibacterium	1.99E−05	0.0032
p__Actinobacteria;c__Actinobacteria;o__Actinomycetales;f__Microbacteriaceae	7.19E−06	0.0032
p__Actinobacteria;c__Actinobacteria;o__Actinomycetales;f__Nocardiaceae;g__Rhodococcus	1.99E−05	0.0032
p__Proteobacteria;c__Alphaproteobacteria;o__Rhodospirillales;f__Acetobacteraceae;g__Roseomonas;s__massiliensis	1.51E−05	0.0032
p__Proteobacteria;c__Alphaproteobacteria;o__Rhizobiales;f__Hyphomicrobiaceae;g__Devosia	1.99E−05	0.0032
*Amplicon sequence variants of adults gut* p__Bacteroidetes;c__Bacteroidia;o__Bacteroidales;f__Rikenellaceae	0.0013	0.039
p__Firmicutes;c__Erysipelotrichi;o__Erysipelotrichales;f__Erysipelotrichaceae	0.0013	0.039
p__Firmicutes;c__Clostridia;o__Clostridiales;f__Ruminococcaceae;g__Clostridium;s__methylpentosum	0.00013	0.0063
p__Firmicutes;c__Clostridia;o__Clostridiales;f__Mogibacteriaceae	5.18E−05	0.0028
p__Tenericutes;c__Mollicutes;o__RF39	4.51E−05	0.0025
p__Bacteroidetes;c__Bacteroidia;o__Bacteroidales;f__Bacteroidaceae;g__Bacteroides	2.12E−05	0.0014
p__Firmicutes;c__Clostridia;o__Clostridiales;f__Lachnospiraceae;g__Dorea	2.12E−05	0.0014
p__Bacteroidetes;c__Bacteroidia;o__Bacteroidales	1.78E−05	0.0014
p__Bacteroidetes;c__Bacteroidia;o__Bacteroidales;f__Porphyromonadaceae;g__Parabacteroides	8.69E−06	0.001
p__Verrucomicrobia;c__Verrucomicrobiae;o__Verrucomicrobiales;f__Verrucomicrobiaceae;g__Akkermansia	1.15E−06	0.00018

**Fig. 2:**
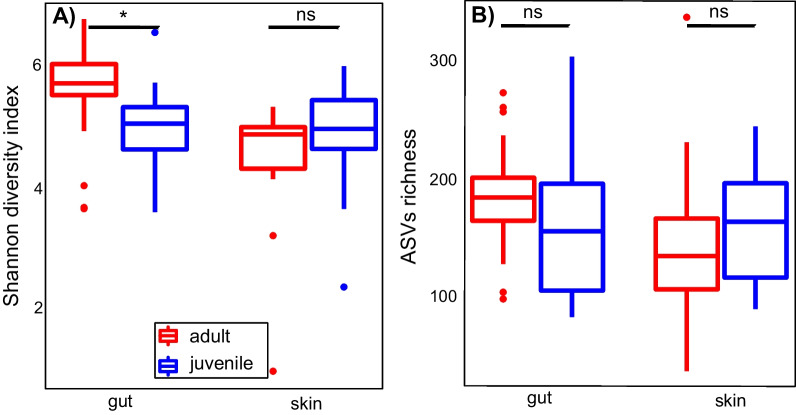
Shannon diversity index and observed ASVs for the skin and gut microbiomes across life stage in *H. squalostoma*. **A** The ANOVA analysis indicates that adults gut diversity is significantly higher than juveniles’ (F_(1,23)_ = 5.25, *p* = 0.03, n = 24) whereas their skin is not (F_(1,24)_ = 0.9, *p* = 0.33, n = 25). **B** No significant differences of observed ASVs was detected between adults and juveniles on the skin (F_(1,23)_ = 0.27, *p* = 0.6) or gut (F_(1,24)_ = 1.78, *p* = 0.18)

**Fig. 3:**
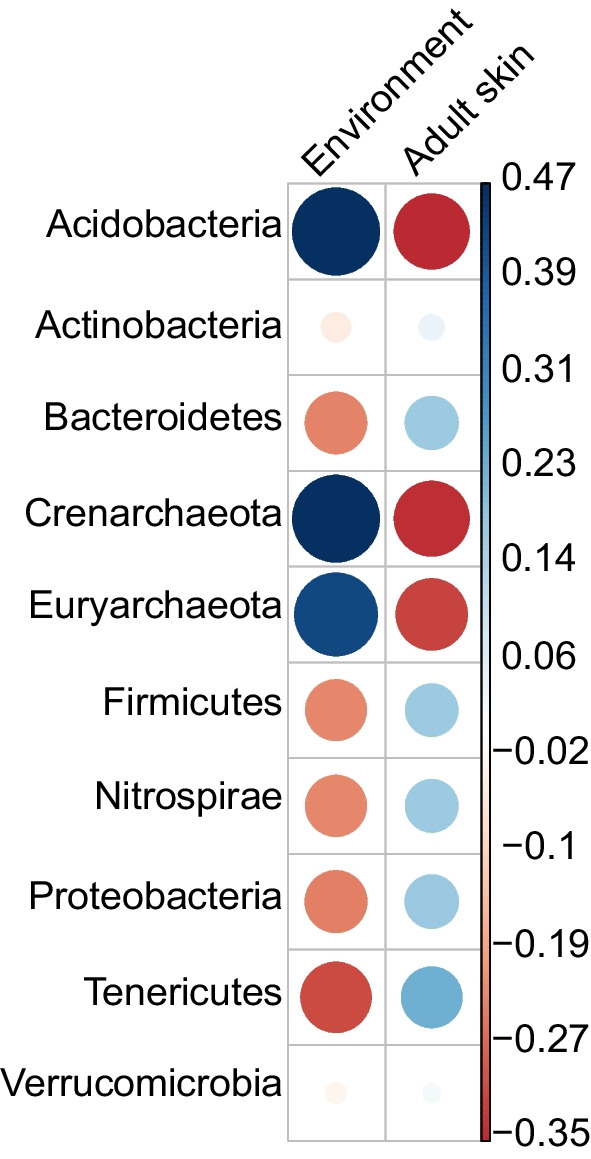
Relationship between the skin bacteria community of adults *H. squalostoma* and the environmental samples evaluated using the Pearson chi-square. The blue color indicates positive associations whereas brown color are negative. The darker the color the stronger the relationship. Note the mismatch of color between adults skin and their environment

The gut core bacteria community comprised ten phyla dominated by Firmicutes and Bacteroidetes (Table 1). The dominant gut bacteria families were Bacteroidaceae, Lachnospiraceae, Mogibacteriaceae, Porphyromonadaceae, Rikenellaceae, Ruminococcaceae, and Verrucomicrobiaceae as well as two other unidentified lineages in the phyla Bacteroidetes and Tenericutes. The most abundant families were Porphyromonadaceae and Mogibacteriaceae, and the most abundant genus, *Parabacteroides* (Porphyromonadaceae). As observed for the skin, the diverse bacteria community of the gut of adult *H. squalostoma* was not dominated by a single genus, and the dominant genera differ from those of the skin of frogs [46]. Shannon diversity indices indicate that the diversity of adult gut communities was significantly higher than those of juveniles, but the diversity of their skin did not differ (Fig. 2). These results were mirrored by those of our calculation of beta diversity which indicated that adult and juvenile skins were similar, contrary to their guts that were significantly different (Additional file 2: Fig. S2). There was no significant difference of observed ASVs between adults and juveniles in either the skin or the gut (Fig. 1). Our investigation of the relationships between the skin communities of adult *H. squalostoma* and the environmental samples found little evidence for an association between these. The Pearson chi-square test indicated strong positive associations between environmental samples and bacteria phyla such as Acidobacteria, Crenarchaeota, and Euryarchaeota, but all of these were only minimally represented in adult skin bacteria communities (Fig. 3).

### Skin and gut bacteria communities of females and males

**Fig. 4:**
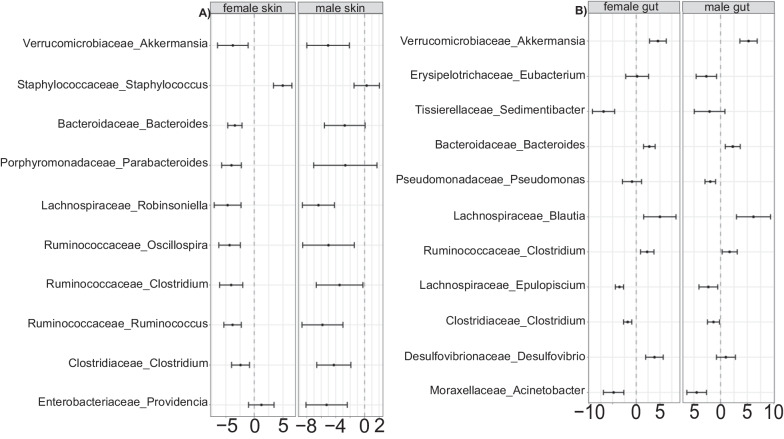
Diffenrentially abundant bacteria families and genera on the skin and in the gut of females and males *H. squalostoma*. Note that for this analysis juveniles skin and gut samples were respectively used as control which allowed us to evaluate differential abundant bacteria with respect to life stage. **A** The shift in abundance of bacteria taxa on the skin of males relative to that of females is remarkable, contrasting with the trend in the gut of females which only shows a mild shift. **B** The gut of males also shows a somewhat mild shift but well noticeable. The results are presented in the form of mean (black dot) with 95% confidence interval represented by the bars

There were sex-related differences in bacterial prevalence with some taxa exclusively encountered in either females or males, and some of these bacteria specific to one sex had high relative abundance. Only male skin was colonized by the bacterial phylum Nitrospirae but lacked bacteria in the families Microbacteriaceae, Gordoniaceae, and Sphingomonadaceae that were all present on the skin of females (Additional file 3: Fig. S3). In our PCoA plot (particularly along the second axis), the skin of females and males formed distinct bacterial clusters (Additional file 4: Fig. S4) though PERMANOVA found these not to be significant. Female skin is dominated by the families Sphingobacteriaceae, Weeksellaceae, Comamonadaceae, and Enterobacteriaceae whereas male skin was primarily colonized by Moraxellaceae and Bacillaceae. Male and female skin also shared bacteria in the phyla Actinobacteria (Micrococcaceae and Sanguibacteraceae) and Proteobacteria (Brucellaceae). Our *corncob* analysis supported these sex-related dissimilarities of the skin microbiomes and found that this pattern was driven by two bacterial taxa: *Staphylococcus sciuri* (Firmicutes) and an unidentified species in the genus *Providencia* (Proteobacteria) (Fig. 4). A similar pattern where the host bacteria community is dominated by taxa that differ between sex was also observed in the gut (Fig. 4).

Our GLMs (Additional file 8: Table S1) detected the effects of sex on the skin and gut of adults *H. squalostoma* for several of the ASVs. Female microbiomes were highly enriched in these ASVs. Though there was an interaction between sex and ẟ^15^N on the skin, the effect of sex alone was highly significant (*p* = 0.0071, df = 1) for the bacteria *Staphylococcus sciuri* (Firmicutes); males showed a 15% decrease in abundance of this species. The sex effect in the gut was more pronounced, and we found that unidentified bacterial species in the genera *Clostridium* and *Coprococcus* (both Firmicutes) as well as *Desulfovibrio* (Proteobacteria) were all significantly more abundant in females (respectively *p* = 0.02, df = 1; *p* < 0.001, df = 1 and *p* = 0.03, df = 1). Similarly, as observed in the skin, the proportions of these bacteria in the male gut were 13.5% less for the genus *Coprococcus* and 49% less for the genus *Desulfovibrio*.

Sex-related differences were also observed throughout the gut subtypes. Female foreguts and midguts were significantly richer and more diverse than those of males (foregut: K–W *χ*^2^ = 40.107, *p* < 0.001, df = 1; midgut: K-W *χ*^2^ = 9.2, df = 1, *p* = 0.002), with 1.2% of ASVs unique to males and as many as 14.7% unique to females. We did not detect any differences between the female and male distal guts (K–W *χ*^2^ = 0.37, df = 1, *p* = 0.53), and the numbers of unique features for females (4.3%) and males (0.6%) were far fewer than observed in the foregut or midgut. In general, female guts were enriched in Firmicutes with an excess in abundance relative to males that ranged from 28 to 57%. Our *corncob* analysis identified seven differentially abundant bacterial taxa in all three gut subtypes including three in foregut, three in mid gut, and one in distal gut. There was only one shared differentially abundant bacterial taxon between foregut and midgut (genus *Desulfovibrio*, Proteobacteria), and none between the distal gut and either the foregut or midgut.

### The effect of maternal status on skin and gut colonization

**Fig. 5:**
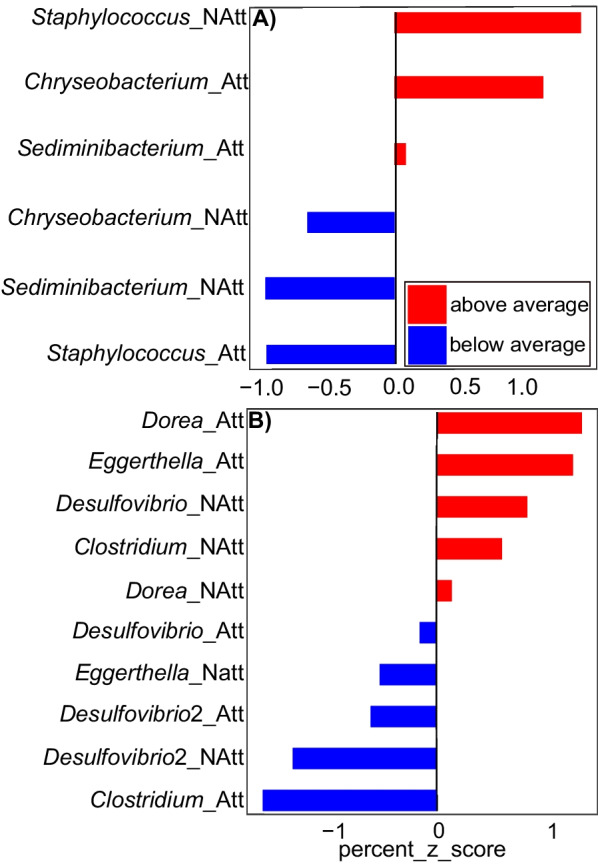
Relationship between females reproductive status and significantly expressed bacteria taxa recovered by the corncob analysis. **A** The skin of mothers (Att) and non mothers (NAtt) harbor bacteria communities with a pattern of expression that differ in its direction (above average or bellow average) and magnitude. Bacteria genus *Staphylococcus* is one of the main driver of this difference. **B** The gut of mothers and non mothers show a similar decoupling of bacteria that are selectively expressed whereby bacteria genera Dorea and Eggerthella are highly expressed in mothers whereas the genus *Desulfovibrio* has a dicreased expression in non mothers

Our *corncob* analysis found three significant differentially abundant bacterial taxa between attending and non-attending females. The skin of attending mothers was significantly enriched in bacteria belonging to the genera *Sediminibacterium* and *Chryseobacterium* (Fig. 5). Bacteria relative abundance was also higher in attending mothers. We recovered 18.7% unique ASVs including the presence of the phyla Verrucomicrobia that was not present in the skin of non-attending mother. The differences in the gut bacteria communities were even more pronounced and our *corncob* analysis found seven significant differentially abundant ASVs between attending and non-attending females (Fig. 5).

### Skin and gut microbiome across life stage

Our *corncob* analysis revealed that juvenile and adult guts shared many more ASVs than their skins (23 vs. 16). The juvenile skin bacteria community was remarkably similar to that of females (Fig. 3), largely resulting from the abundance of the genus *Staphylococcus* (Firmicutes). Female and juvenile skins further resembled one another by sharing several bacteria taxa including families Verrucomicrobiacea (Verrucomicrobia) and Nocardioidaceae (Actinobacteria) and Erysipelotrichaceae (Firmicutes). This similarity was only observed between juveniles and mothers but not with non-mothers which lacked all bacteria shared by juveniles and females (Additional file 5: Fig. S5). Skin bacteria that were specific to juveniles included the family Christensenellaceae (Firmicutes). Differences across life stages were more evident when considering the adult foregut and distal gut (Additional file 6: Fig. S6). The adult distal gut lacked the bacteria phylum Synergistetes, which was present in both the adult midgut and juvenile gut. Juvenile and adult guts shared four ASVs with a unique pattern of abundance: moderate to low in most adult guts but high in all juvenile guts, in which they were significantly abundant (Additional file 6: Fig. S6). These shared ASVs represent two genera: *Epulopiscium* (Lachnospiraceae) and *Clostridia* (Clostridiaceae, Firmicutes). 

### Microbiome transmission between juveniles and attending females

**Fig. 6:**
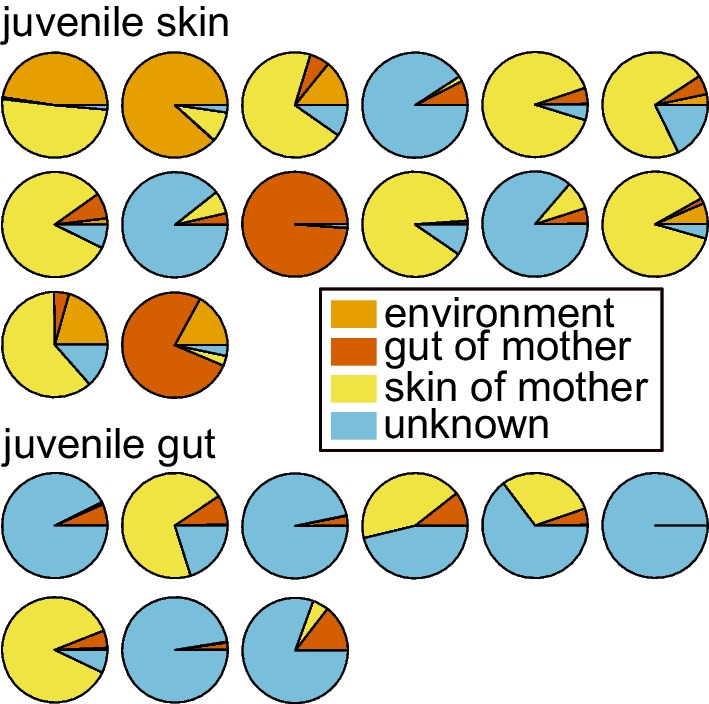
The skin and gut microbiome community of *H. squalostoma* juveniles in relation to their bacteria sources assessed by SourceTracker. Sources include the skin and gut of mothers and the environmental samples (leaf, water and soil) at the site where caecilian sampling took place). Note that the skin of most juveniles is dominated by the skin of mothers which also represent an important proportion of the gut of some juveniles

**Fig. 7:**
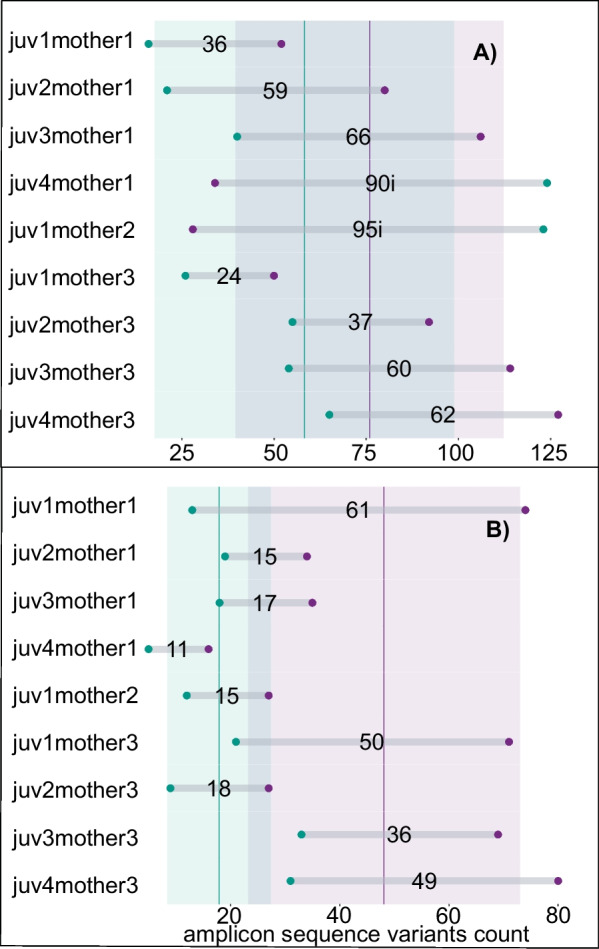
Raw count of ASVs shared by the skin and gut of juveniles and their mothers. Vertical bars indicate the standard error and numbers followed by 'i' refer to samples for which the contribution of mother gut to juveniles skin was higher than that of mother skin. ASVs shared between the skin of juveniles (**A**) were higher than those shared the gut (**B**) of their mothers (Pearson *χ*^2^ = 201.54, *p* < 0.001, df = 8)

The sourcetracker analysis indicated that the skin and gut microbiomes of attending mothers are a source for the juvenile skin and gut bacteria communities (Fig. 6). Without exception, all juveniles shared some proportion of their skin and gut communities with those of their respective mother. Juveniles shared between 4 and 19% of the ASVs of their skin communities with their mother’s skin (mean = 76 ± 36; min = 28; max = 127), 3–20% of their gut communities with their mother’s gut (mean = 18 ± 9; min = 5; max = 33), 3–24% of their skin bacteria with their mother’s gut (mean = 58 ± 40; min = 16; max = 154), and 4–18% of their gut communities with their mother’s skin (mean = 48 ± 25; min = 16; max = 80). To further explore this pattern, we removed juveniles that were missing either gut or skin samples, thus retaining nine juveniles (n = 9) that we compared to their mothers. We found that, on average, a mother’s skin shared significantly (Pearson χ^2^ = 201.54, *p* < 0.001, df = 8) more ASVs with their respective juveniles’ skins (mean = 68 ± 35; min = 28; max = 127) than guts (mean = 56 ± 50; min = 9; max = 80). (Fig. 7). Only two of the nine juveniles (22%) shared more ASVs with their mother’s gut than skin. The abundance of these ASVs was two times larger than the average abundance of ASVs shared between their respective mother’s gut and any other juvenile gut samples. In addition, the corresponding ASV abundances that these juveniles’ skins shared with their mother’s skin were the lowest of all skin samples. Similar to the juvenile skins that were dominated by ASVs in their mother’s skin, juvenile guts shared higher ASV abundances with mother’s skin than gut (though Pearson chi-square test did not find this difference to be significant; χ^2^ = 10.5, df = 8, *p* = 0.23; Fig. 7). On average, mother’s skins (mean ASVs = 62 ± 26; min = 27; max = 103), but not guts (mean ASVs = 38 ± 19; min = 14; max = 68), shared a more abundant bacteria community with juvenile skin and gut. However, the importance of the mother’s skin in contrast to the gut (Additional file 7: Fig. S7) was not significant (*p* = 0.08, t = − 1.93, df = 8). The results of our sourcetracker analysis indicated that the dominant gut bacteria in most juveniles originated from an unknown source that accounted for 63–100% (mean = 68.9% ± 33.3) in 70% of sampled juveniles. This unknown source shared bacteria equally with the skin of most juveniles and dominated the contribution of the environment in most samples (Fig. 6). The environmental contribution to the gut was the least important of all sources for juvenile bacteria sources both on their skin and gut, and its contribution to the skin was negligeable (mean = 0.1% ± 0.3).

**Fig. 8:**
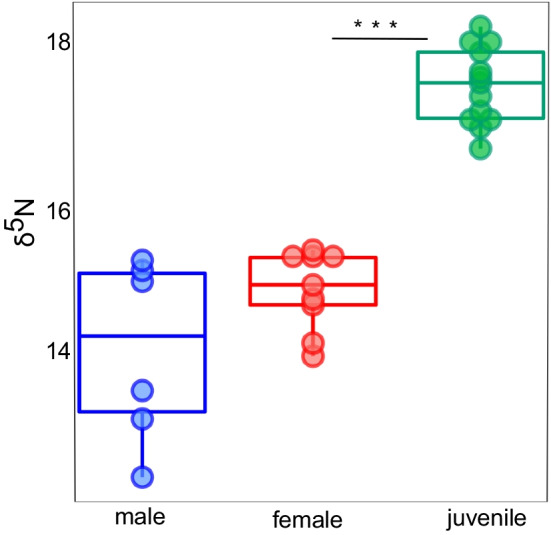
Nitrogen isotopic content for the skin of juveniles and adults *H. squalostoma*. The isotopes values for juveniles are significantly higher (ANOVA, F_(2,25)_ = 65.54, *p* < 0.001) than those of either males or females. These values are in the range of content expected between preys and their predator which suggests that juveniles feed at a higher trophic level relative to adults. This trend reinforces the observation that juveniles feed on the skin of their mother

The results of our one-way ANOVA on the relationship between stables isotopes and life stage (Fig. 8) showed a significant difference between juveniles and adults (F_(2,25)_ = 65.54, *p* < 0.001). A post-hoc Tukey test showed that juvenile ẟ15N values were significantly higher than those of males and females respectively, but adults did not differ significantly by sex (Additional file 9: Table S2). Values were higher for juveniles (range 16.6–18.15‰, mean = 17.4 ‰ ± 0.5) than adults (range 12.5–15.3‰ mean = 14.5‰ ± 0.9).

## Discussion

The skin and gut bacteria of adult *H. squalostoma* are typical of most other vertebrates by being dominated by few bacterial phyla, including Actinobacteria and Proteobacteria on the skin and Firmicutes and Bacteroidetes in the gut [47]. However, we found notable differences between the core skin bacteria community of adult *H. squalostoma* and those of other amphibians. The dominant bacterial taxa on the skin of adult *H. squalostoma* (Acetobacteracea, Brevibacteriaceae, Brucellaceae, Comamonadaceae, Hyphomicrobiaceae, Microbacteriaceae, Nocardiaceae, *Comamonas*) do not include the Pseudomonadaceae and Moraxellaceae or the genus *Pseudomonas* that are often found to dominate the core skin microbiome of frogs [15, 48] and salamanders [13, 49]. Likewise, the core gut bacteria community of adult *H. squalostoma* was dominated by the phyla Verrucomicrobia and Tenericutes, which rarely dominate the core gut microbiome of other amphibians [50, 51]. In addition, the dominance of the gut community of *H. squalostoma* by the genus *Parabacteroides* is unusual among vertebrates. Whether these patterns in the skin and gut microbiomes are common among caecilians requires evaluation in future studies. We also found that adult gut communities showed significantly more diversity (i.e., Shannon diversity index) than those of juveniles, a pattern commonly found in mammals and especially in the human gut microbiome [31, 32]. In contrast, juvenile skin bacterial communities were somewhat more diverse than adults, though this difference was not statistically significant.

### The effect of parental care on microbiome transmission

Our results indicate that parental care contributes to microbiome transmission between mothers and juveniles in *H. squalostoma*, an oviparous direct-developing and skin-feeding caecilian. The results of our source-sink analysis (Fig. 6) indicate that most juveniles derive a portion of their skin and gut microbiomes from the skin and gut bacteria of attending adult females, including skin-to-skin, gut-to-gut, and cross-colonization between skin and gut. Skin-to-skin colonization suggests that frequent skin contact and proximity of the mother and juveniles is important for bacterial colonization of juvenile skin. During attendance of eggs and juveniles, female *H. squalostoma* coils around juveniles with the entire clutch forming a compact unit [41], likely promoting skin-to-skin colonization. Our analysis using sourcetracker revealed gut-to-gut colonization that might result from maternal fluid provisioning to juveniles. Maternal fluid delivery is hypothesized to be shared trait for oviparous direct-developing caecilians [40], though has yet to be observed in *H. squalostoma*. It is also possible that other mechanisms, such as consumption of the mother’s feces (whether intentional or not), could also result in gut-to-gut colonization. Our source-sink results revealed that adult skin is also a source of bacteria for the juvenile gut, indicating that feeding on their mother’s skin is a likely source of both microbiome transmission and nutrition in juveniles. This is also evident in the difference in ẟ^15^N values between adults and juveniles, with values being higher in juveniles. Our results suggest that parental care plays a role in microbiome transmission in *H. squalostoma,* similar to that found in other vertebrate taxa such as fishes [21], birds [20], and mammals [19], including most notably humans [25, 29], in which it is seen as a mechanism for maximizing offspring fitness and survival [52].

Our observations in this caecilian species provide the strongest evidence to date of the role of parental care in fostering vertical microbiome transmission in an amphibian. Previous studies of microbiome transmission associated with parental care among oviparous and direct-developing frogs and salamanders have found the parental microbiome to have little or no impact on juvenile microbiomes [36, 45, 53]. For the other amphibian species studied to date, this pattern might not be surprising as egg attendance has been described as largely facultative. In the plethodontid salamander *Hemidactylium scutatum*, for example, some attending females abandon their eggs [54, 55]. Eggs desertion is not only common in this species but can lead to loss of embryos due to desiccation, predation, and fungal infection [45, 54]. Similar patterns of eggs desertion are common in *H. colymbiphyllum* in which attending males can sometimes be found in close proximity to the eggs but without physical contact [36, 56]. In our study, female *H. squalostoma* were found while attending their juveniles and tightly coiled around them [41, 57]; it is unknown whether females might leave and return to their clutch. We interpret these instances of “committed” attendance in *H*. *squalostoma* as opportunities for skin-to-skin microbiome transfer via direct skin contact, also supported by our findings that beta diversity was very similar between adult and juvenile skins within families. This obligate behavior likely favors microbe maintenance by the mother throughout attendance, though we do not know exactly how long parental care lasts in *H. squalostoma*. Moreover, skin-feeding provides an additional opportunity for vertical microbiome transmission to occur in *H. squalostoma* [41]. Ours is the only study to date of microbiomes in an amphibian in which an adult provisions offspring, though other known species provide opportunities for future research [58].

### Microbiome colonization and the environment

We found limited evidence for the role of the environment in shaping the skin microbiome of *H. squalostoma*. Both the Pearson chi-square and sourcetracker analyses indicated that the environment (soil, water, and leaves) contributed little to the skin bacteria community of females and males in our sample. Our finding contrasts with our prediction that living and moving through the soil would shape the skin microbiome of soil *H. squalostoma* [44]. We know of no other studies of the microbiome in burrowing vertebrates, and our observations likely provide only a glimpse of how the skin microbiome can relate to subterranean habitats. In contrast, much more is known of the microbiome of earthworms, for which ecology is thought important for explaining differences in the microbiome. In earthworms that are strictly burrowing (endogeic), active above ground (epigeic), or active both above and below ground (anecic), species share abundant bacteria communities with their respective microhabitats [59]. In contrast, our results for *H. squalostoma* show relatively little impact from the microbial community of the surrounding soils, suggesting that processes of microbial colonization in subterranean animals may differ substantially.

Of all sources examined in our study, bacterial taxa with unknown sources were dominant in most juvenile guts and the most frequent and often dominating source for juvenile skins. The importance of these unknown sources suggests that some key bacteria taxa are either undetectable by our analysis or simply missing. One potential missing source is the arthropod community that constitutes the diet of these caecilians [57]. In this study, we did not examine juvenile gut contents nor did we sample the microbiome of co-occurring arthropod communities. In the future, sampling the microbiomes of these sources may provide insights into the missing bacteria sources and help to understand the role of prey items in structuring the juvenile skin and gut microbiome communities. Juvenile *H. squalostoma* begin feeding early in life [57] and their interactions with prey items (e.g. [60]; see Fig. 1a, [61]) may lead to the diverse juvenile microbiome communities in the skin and gut. Another possibility is that these bacteria taxa could not be correctly classified in our analyses and thus appear as coming from unsampled sources.

### Microbiome across life stages

Life stage was a key factor influencing both skin and gut microbiome composition and abundance in *H. squalostoma*. The juvenile gut communities were markedly different from those of adults. This is similar to observations from other vertebrates such as fishes [21] in which these differences are due to reduced gut bacteria richness and diversity in juveniles. The results of our *corncob* and *ANCOM* revealed a lower richness in the juvenile skin and gut microbiomes. Further, *ANCOM* revealed only four significant differentially abundant bacteria in juvenile guts, which are likely the primary colonizers of the juvenile gut. That these primary colonizers are not as dominant in adults indicates that they are probably outcompeted by other bacteria taxa that become more dominant in later life. Adults gut communities were dominated by the bacterial families Erysipelotrichaceae, Ruminococcaceae and Rikenellaceae, rather than the Actinomycetales and three species in the family Lachnospiraceae found in juveniles. Similar age-related differences and dynamics in microbial communities are known to occur in other vertebrates including fishes and mammals. In humans, the newborn skin and gut are seemingly sterile before birth and these niches are colonized during vaginal birth by opportunistic bacteria (primary colonizers) from the mother’s skin and gut [29, 30]. In the skin-feeding fish *Symphysodon aequifasciata* [21], bacteria remodeling with age occurs in the gut of juveniles that feed on the mucous of the parents [21]. Because of our limited sampling of ontogenetic stages, we cannot evaluate the dynamics of the gut microbiome in *H. squalostoma*, such as when in an individual’s life it achieves the adult gut microbiome.

We found that gut microbiome varied more in adults than juveniles, and this may reflect their more diverse diets. In contrast to the diets of juveniles that contain just ants, earthworms, and the mother’s skin, adults consume at least ten different invertebrate prey types [57]. This diversity of prey in the adult diet likely generates an equally diverse bacterial community in the adult gut [2].

## Conclusion

Our study provides strong support that parental care in the caecilian *Herpele squalostoma* promotes skin and gut microbiome transmission from attending mothers to the skin and gut communities of their juveniles. The mother’s skin plays a particularly important role, likely due to both close contact between mother and offspring as well as their feeding on the mother’s skin. Future field research may provide observations on the mechanisms of microbiome transfer from the mother’s gut to the juvenile’s gut. Our analyses suggest that the differences in parental care and investment among amphibians can lead to distinct differences in vertical transmission of microbiomes. Parental care that is limited to passive or facultative attendance, including occasional physical contact between the parent and their eggs or offspring may lead to minimal vertical transfer, such as has been found in several frog and salamander species [62–64]. In contrast, strategies that include extended skin-to-skin contact of neonates and nutritional provisioning of offspring [65, 66] likely promotes vertical transmission of the skin and gut microbiomes, as we found for *H. squalostoma*. The wide diversity of both life histories and parental care strategies among amphibians provides ample opportunity for future studies that may find further evidence for modes of microbiome transmission among vertebrates.

## Methods

### Field work and microbiome sampling

Our samples of caecilians were obtained in southeastern Cameroon (Central Africa) in the buffer zone of the Dja Biosphere Reserve (DBR) that encompasses 526 km^2^. The DBR is part of the Congo Basin and comprises extensive primary tropical rainforest that is semideciduous [67]. The DBR is bisected by a series of drainages, most of which converge to the Dja River that borders the DBR to the northeast, west, and south [68]. The area is home to sparse settlements of Bantou and Baka peoples. We sampled in Bifolone at a site characterized by primary forest that extends along a mild slope adjacent to a dense patch of mixed species of grasses (ranging in height from 0.5 to 2 m) leading to a swampy area terminating in a stream (~ 1 m wide) that flows into the Dja River (~ 50 m to the north). There were several seepages from the soil along the edge of the forest, causing the predominantly claylike soil to be wet or damp. Sample collection took place during two digging events on 1 and 8 June 2018 carried out between 10 AM and 3 PM. Five people digging with hoes actively searched for caecilians in the top 10–15 cm of soil; in total, there were ~ 30-person hours of effort searching ~ 68 m^2^ of the soil surface. All specimens of *H. squalostoma* were kept in individual and sterile perforated plastic containers. When a family of *H. squalostoma*—comprised of an attending mother and juveniles—was encountered, they were kept together in a single larger container. Specimens were removed from the container by a person wearing sterile nitrile gloves that were exchanged between specimens. Because each mother is wrapped around her offspring—and thus in close skin-to-skin contact—gloves were not replaced between individuals of a single family. At each of our two sampling events, we took three environmental samples comprising soil, water from a seepage, and leaf within 10 m radius of the sampling spot. To sample the soil and the water from the seepage, a swab (MW 113, Medical Wire, UK) was dipped in and twisted; for the leaf, we picked a leaf ~ 10 cm above the soil to which we applied 15 swab strokes on both surfaces. At most 24 h after caecilians were obtained, all individuals were processed for skin and gut microbiome following standard protocol [15]. We first rinsed animals with 50 mL of filtered (pore size, 0.2 μm) bottled water to remove transient bacteria on their skin. For the skin microbiome, we applied 30 swab strokes on the skin from head to the rear but avoiding the cloaca, gently rotating the animal and the swab as we sampled. Subsequently, the animal was euthanized in a bath of 0.2% methanesulfonate benzo-tricaine (MS 222) [69] and its length in millimeters and weight in grams were recorded. We measured the animal’s length using a measuring tape and its weight on a laboratory scale. We then sampled for the gut microbiome, beginning by using forceps, scalpels, and scissors to make a longitudinal incision of the animal’s ventral side of the belly (from bellow the heart to above the cloaca) and we then gently opened the gastrointestinal (GI) track longitudinally. To sample the gut, a swab (MW 113) was dipped and rotated in the GI track of the animal. For adult caecilians, we took three gut samples: the foregut at the proximal end of the GI track below the stomach; the middle gut; and the distal gut approximately 2 cm from the cloaca. All environmental, skin, and gut samples were preserved in a solution of filtered sterilized (0.22 µm, CELLTREAT) 20% glycerol (Fisher Scientific). Following collection, microbiome samples were immediately stored suspended in a liquid nitrogen dry shipper. We took a sample of liver tissues of euthanized *H. squalostoma* and stored these in RNALater for other studies. Euthanized animals were fixed in formalin for 24 h, washed with water, and transferred to 70% ethanol for storage. We maintained systematically high hygienic conditions throughout field collecting and microbiome sampling by wearing gloves and exchanging these between specimens and during microbiome sampling. Likewise, between each sampling event, forceps and scissors were submerged in 10% hydrogen peroxide for a minimum of 7 min and then rinsed with filtered bottled water before reuse [70]. All caecilian specimens and microbiome samples were later shipped to the US where microbiome samples were transferred to a − 80 °C freezer until processing. All preserved specimens and tissue samples were deposited in the Herpetology Division of the Florida Museum of Natural History at the University of Florida (Gainesville, Florida, USA).

We determined the sex for adult *H. squalostoma* by examining the gonads of preserved specimens. Males were identified by the presence of testes, which are paired, elongated, granular, and white/pale soft organs that are found along the long axis of the body and interconnected by a thread-like duct [71]. The ovaries of females are paired, ovoid, sac-like, and parallel to the long axis of the body and contained oocytes at different stages of maturation.

### DNA extraction, PCR amplification, and sequencing

We extracted genomic DNA from swabs using the DNeasy Qiagen kit for blood and animal tissue following the manufacturers recommended protocol for extracting gram-positive bacteria. The swabs were incubated during two consecutive steps. We added 180 μL of lysozyme lysis buffer and incubated at 37 °C for an hour. Then 25 μL of proteinase K and 200 μL of AL buffer were added to the swab and incubated at 70 °C for 30 min. PCR amplification of extracted DNA targeted the V4 hypervariable site of the 16S rRNA. We used the set of primers recommended by the Earth Microbiome Project: the 515F and 806R primers [72]. We used 1 μL of extracted DNA template in a total reaction mix of 25 μL which comprised 13 μL of PCR grade water, 10 μL of PCR master mix and 0.5 μL of forward primer (concentration: 10 M) and 0.5 μL of reverse primer (10 M) as specified by the EMP. The temperature profile of the thermocycler was as follows: 94 °C for 3 min; 35 cycles of 94 °C for 45 s, 50 °C for 60 s and 72 °C for 90; 72 °C for 10 min and lastly a 4 °C hold. All PCRs were run in duplicate alongside a no-template control, and the resulting products were pooled per sample. DNA verification in each sample amplicon was conducted by running an agarose gel in which a readily visible band was indicative of the presence of DNA. We conducted library preparation at the University of Massachusetts, Boston where sequencing was on an Illumina Miseq platform.

### Sequence, processing, and bioinformatics

All bioinformatics analyses were conducted using the Quantitative Insight Into Microbial Ecology (*QIIME* 2) workflow (version 2020.8) and using several of its built-in plugins to process our Illumina Miseq sequence reads [73, 74]. Forward reads were demultiplexed and barcodes and adapter removed while assigning reads back to samples. Then sequences were denoised using QIIME2 q2-DADA2 [75]. The DADA2 plugin is capable of identifying true and false sequence differences with a margin as small as one base-pair and yields a high-resolution table of amplicon sequence variants (ASVs, amplicon sequence variants or sequence reads), which is much more reliable than the standard table of OTUs (Operational Taxonomic Units) that is prone to spurious sequences [75]. After denoising sequences, taxonomy was assigned using the Greengenes pretrained classifier (g_13) [76, 77].

### Stable isotopes sampling

We measured ẟ^15^N from our preserved specimens of *H. squalostoma*. Fluid-preserved specimens exposed to formalin or ethanol were once deemed problematic for collecting isotopic data over concerns that preserving chemicals can alter the signature ratios of carbon and nitrogen isotopes of the processed samples [78], but more recent studies found the effect of preservation to be negligeable [79, 80]. This has led to a growing interest in using fluid-preserved specimens to evaluate trophic relationships among communities of species that are difficult to sample in the wild (e.g., [81, 82]), such as caecilians. For our stable isotopes analysis, we took skin and muscle fragments at mid-body of adult and juvenile specimens of *H. squalostoma*, all of which had been fixed in buffered-formalin and stored for approximately 2.5 years in 70% ethanol. This standardized preservation and sampling technique should minimize variation of stable isotope ratios among samples due to alterations of isotope signature [83, 84]. Sampled skin and muscle were soaked in DI water for five days and transferred to an oven for drying at 37 °C for 24 h. Dried samples were weighed to the nearest 0.0001 mg and packed in tin capsules. The ẟ^15^N was measured in the Geology Department of the University of Florida on a Thermo Electron DeltaV Advantage isotope ratio mass spectrometer coupled with a ConFlo II interface linked to a Carlo Erba NA 1500 CNHS Elemental Analyzer.

### Statistical analyses

Statistical analyses were conducted using both *QIIME* and the R statistical software (version 4.0.3) [85]. *Herpele squalostoma* samples were evaluated for patterns that may be attributed to differences in microbiome type (skin and gut, including subtypes such as foregut, mid gut, and distal gut of adults), life stages (adults and juveniles), adult sex (females and males), and maternal status (non-attending females and attending females). We computed the core microbiome of adults *H. squalostoma* to determine the specific microbiome communities associated with the skin and gut samples. For this analysis, we used COREMIC ([86]; http://coremic2.appspot.com), a web application that converts bacteria relative abundance into presence/absence data then uses the Fisher Exact Test to evaluate whether there is a significant relationship between the observed bacteria communities and a given niche (e.g., skin). The *p* values were corrected for multiple-testing using the Benjamini–Hochberg procedure [87]. We computed microbiome differential abundance using both the analysis of composition of microbiomes (*ANCOM*) [88, 89] and *corncob* [90], an R package that uses a beta-binomial distribution to evaluate the relationships between bacteria relative abundance and covariates of interest. These two techniques are robust across studies and datasets [91] and for the *corncob* analysis our covariates comprised gut subtype (foregut, middle, distal), sex (females and males), reproductive status (attending mother and non-attending mother), and life stage (adult and juvenile).

To evaluate the extent to which diet shapes the skin and gut microbiomes of adult female and male *H. squalostoma,* we performed a quasi-Poisson generalized linear model (GLM). This approach can accommodate the overdispersion that is often characteristic of count data [92] as found in an ASV table. We restricted this investigation to the significant differentially abundant ASVs obtained from the *corncob* analysis with sex as a covariate. In this analysis, bacteria relative abundance was the dependent variable and both sex and ẟ^15^N values from the skin were the independent variable. We used a one-way ANOVA to evaluate the significance of this relationship with ẟ^15^N representing the dependent variable while life stage was the independent variable. Another analysis consisted of computing samples group significance (clustering), achieved by applying the robust centered log-ratio transformation to non-zero entries in our dataset [93]. This method is appropriate for compositional data and is reliable for evaluating a microbiome features table [88]. This analysis yielded principal coordinate analysis (PCoA) plots generated by the *DEICODE* tool box and which serves to link specific features in the dataset to beta-diversity ordination in *QIIME* [94]. We then assessed the strength of clusters recovered by our PCoA using a permutation analysis of variance (PERMANOVA). Unless otherwise stated, we evaluated community richness with an approach that combined the results of ASV ranking and *ANCOM.* We visualized ASV ranking with the visualization tool *Qurro* [95, 96] and determined the top and bottom 5% ASVs. We assessed differences in community richness among group of samples with the Kruskal–Wallis (K–W) test [97] and tested for the independence of group of samples using the Pearson chi-square test [98]. All analyses were evaluated for significance using a threshold of alpha = 0.05.

We used R to implement the package sourcetracker [43], a method that employs a Bayesian approach and utilizes the Gibb’s sampler. This method identifies shared bacteria between the host (a sink) and potential reservoirs (the possible sources). For this analysis, we considered the skin and gut microbiome of juveniles to be the sinks and the sources to be the bacterial communities in the environment as well as all gut and skin samples of the attending mother. We expected that for transmission to take place, a given source must share some proportion of its bacteria community with the sink. This analysis allows us to address (1) whether there is vertical microbiome transfer between mother and juveniles, (2) the magnitude of the contribution of the maternal skin and gut in shaping juvenile skin and gut microbiomes, and (3) the role of the environment in microbiome transmission in this caecilian species.

## Supplementary Information

**Additional file 1. Figure S1**. Mean number of sequences recovered by *QIIME* from skin and gut samples of adults and juveniles *H. squalostoma*

**Additional file 2. Figure S2**. Beta diversity across life stage for skin and gut microbiome samples of *H. squalostoma*

**Additional file 3. Figure S3**. Composition and relative abundance of bacteria family on the skin of females and males *H. squalostoma*

**Additional file 4. Figure S4**. Skin bacteria community of females (red) and males (blue) *H. squalostoma* in our samples indicating clustering by sex following axis 2

**Additional file 5. Figure S5**. Composition and relative abundance of skin bacteria of juveniles and females *H. squalostoma* according to their reproductive status

**Additional file 6. Figure S6**. Highly expressed ASVs recovered by *ANCOM* in the gut of juveniles and adults *H. squalostoma*

**Additional file 7. Fig. S7**. Distribution of shared ASVs between the skin and gut of juveniles and their mothers skin and gut.

**Additional file 8. Table S1**. The effect of sex and stable isotopes ratio of nitrogen (ẟ15N) on the relative abundance of skin and gut microbiome of adults *H. squalostoma*

**Additional file 9 Table S2**. Tukey post hoc test indicating differences across life stage of skin nitrogen stable isotope (ẟ15N)

## Data Availability

We deposited the datasets at the NCBI SRA repository under the bioproject (TO BE ADDED AT ACCEPTANCE).
