# Supplementary material for: Parental care contributes to vertical transmission of microbes in a skin-feeding and direct-developing caecilian
Source: Anim Microbiome. 2023 May 15;5:28. doi: 10.1186/s42523-023-00243-x (PMC10184399; doi:10.1186/s42523-023-00243-x)
Supplement: Supplementary file 1 — Additional file 1. Figure S1. Mean number of sequences recovered by QIIME from skin and gut samples of adults and juveniles H. squalostoma [file 42523_2023_243_MOESM1_ESM.pdf]

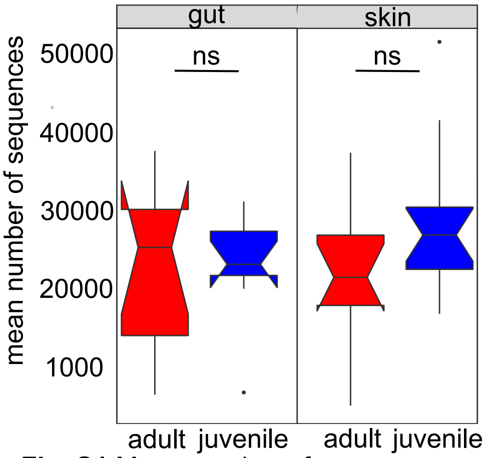

Fig. S1 Mean number of sequences recovered by QIIME from skin and gut samples of adults and juveniles *H. squalostoma*. One way ANOVA did not detect a significant difference of the number of ASVs neither on the skin (p = 0.08) nor on the gut (p = 0.99) samples across life stage.
