# Supplementary material for: Parental care contributes to vertical transmission of microbes in a skin-feeding and direct-developing caecilian
Source: Anim Microbiome. 2023 May 15;5:28. doi: 10.1186/s42523-023-00243-x (PMC10184399; doi:10.1186/s42523-023-00243-x)
Supplement: Supplementary file 2 — Additional file 2. Figure S2. Beta diversity across life stage for skin and gut microbiome samples of H. squalostoma [file 42523_2023_243_MOESM2_ESM.pdf]

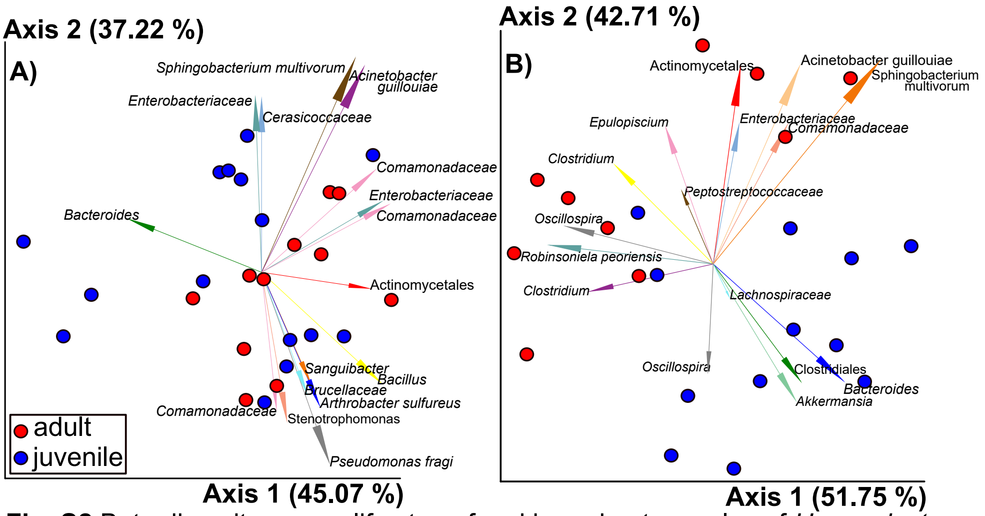

Fig. S2 Beta diversity across life stage for skin and gut samples of *H. squalostoma*. The arrows indicate bacteria taxa driving group clustering and their length is proportional to the magnitude of their effect. Group clustering was evaluated using PERMANOVA with default parameters in QIIME and rarefaction threshold set to 3000. **A)** We failed to detect significant differences between the skin of adults (red) and juveniles (pseudo F = 1.37, p = 0.25. **B)** In contrast, the gut (mid-gut) of adults was signficantly different (pseudo F = 9.6, p = 0.001) from juveniles'. The pairwise samples comparison that employed several beta diversity indices indicated the following: Bray-Curtis (B-C), Pseudo F = 1.68, p = 0.08, df = 24; Unweighted UniFrac (U-U), Pseudo F = 2.36, p = 0.002; Weighted UniFrac (W-U), Pseudo F = 2.2, p = 0.05 for skin samples. For the gut B-C, Pseudo F = 5.57, p = 0.001 df = 21; U-U, Pseudo F = 5.82, p = 0.001; W-U, Pseudo F = 6.76, p = 0.001.
