# Supplementary material for: Parental care contributes to vertical transmission of microbes in a skin-feeding and direct-developing caecilian
Source: Anim Microbiome. 2023 May 15;5:28. doi: 10.1186/s42523-023-00243-x (PMC10184399; doi:10.1186/s42523-023-00243-x)
Supplement: Supplementary file 3 — Additional file 3. Figure S3. Composition and relative abundance of bacteria family on the skin of females and males H. squalostoma [file 42523_2023_243_MOESM3_ESM.pdf]

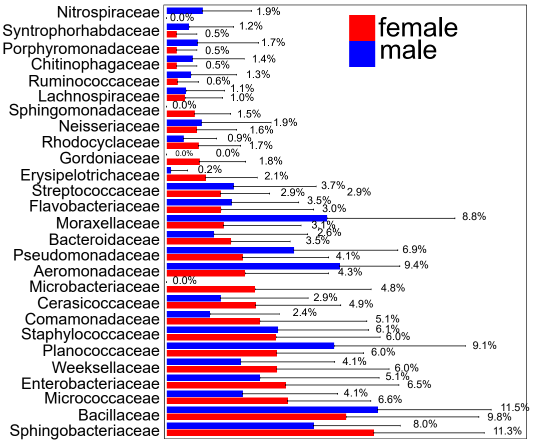

Fig. S3 Composition and relative abundance of bacteria family on the skin of females and males *H. squalostoma*. Bars represent mean ± sd.
