# Supplementary material for: Parental care contributes to vertical transmission of microbes in a skin-feeding and direct-developing caecilian
Source: Anim Microbiome. 2023 May 15;5:28. doi: 10.1186/s42523-023-00243-x (PMC10184399; doi:10.1186/s42523-023-00243-x)
Supplement: Supplementary file 4 — Additional file 4. Figure S4. Skin bacteria community of females (red) and males (blue) H. squalostoma in our samples indicating clustering by sex following axis 2 [file 42523_2023_243_MOESM4_ESM.pdf]

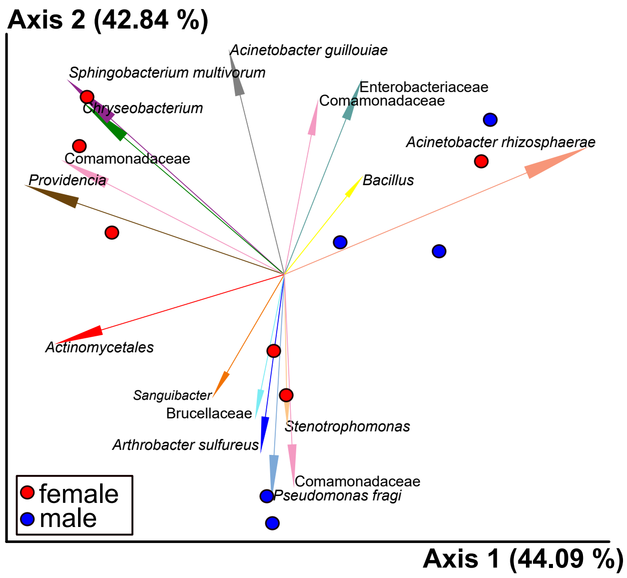

Fig. S4 Beta diversity across sex for skin samples of *H. squalostoma*. Note the clear separation of males and females samples following axis 2 though PERMANOVA failed to detect a significant difference (Pseudo F =1.1, p = 0.12).
