# Supplementary material for: Parental care contributes to vertical transmission of microbes in a skin-feeding and direct-developing caecilian
Source: Anim Microbiome. 2023 May 15;5:28. doi: 10.1186/s42523-023-00243-x (PMC10184399; doi:10.1186/s42523-023-00243-x)
Supplement: Supplementary file 6 — Additional file 6. Figure S6. Highly expressed ASVs recovered by ANCOM in the gut of juveniles and adults H. squalostoma [file 42523_2023_243_MOESM6_ESM.pdf]

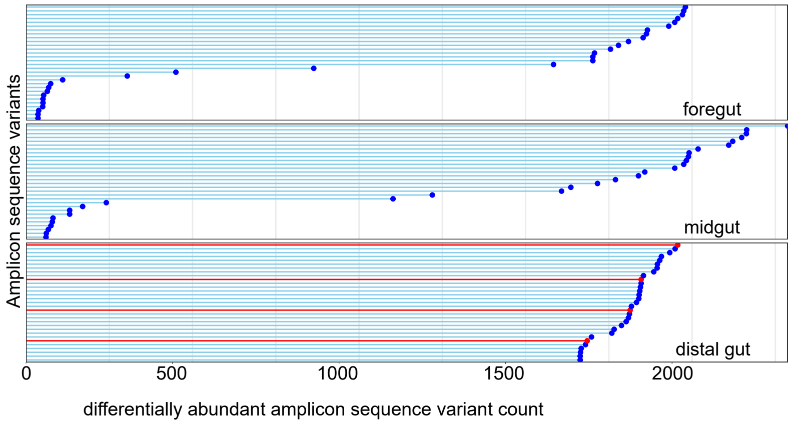

Fig. S6 Significant differentially expressed ASVs recovered by *ANCOM* in the gut of juveniles and adults *H. squalostoma*. ASVs in blue were uniquely recovered from adults foregut (top), midgut (middle) and distalgut (bottom) whereas red ASVs were highly expressed in both adults and juveniles with highest expressions observed in juveniles.
