# Supplementary material for: Parental care contributes to vertical transmission of microbes in a skin-feeding and direct-developing caecilian
Source: Anim Microbiome. 2023 May 15;5:28. doi: 10.1186/s42523-023-00243-x (PMC10184399; doi:10.1186/s42523-023-00243-x)
Supplement: Supplementary file 7 — Additional file 7. Fig. S7. Distribution of shared ASVs between the skin and gut of juveniles and their mothers skin and gut. [file 42523_2023_243_MOESM7_ESM.pdf]

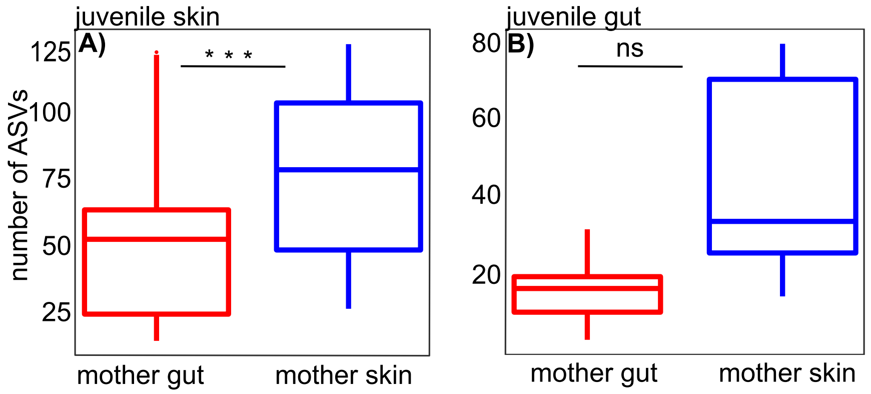

Fig. S7 Distribution of shared ASVs between the skin and gut of juveniles and their mothers. **A)** ANOVA indicates that the skin of mothers shared significantly ($F_{(1,8)}$ = 13.8; df = 8, *p* = 0.004) more ASVs with the skin of juveniles than their guts. **B)** The gut of juveniles shared more ASVs with the skin of mother than the gut but this relationship was not significant ($F_{(1,8)}$ = 1.1; df = 8; *p* = 0.31).
