# Supplementary material for: Parental care contributes to vertical transmission of microbes in a skin-feeding and direct-developing caecilian
Source: Anim Microbiome. 2023 May 15;5:28. doi: 10.1186/s42523-023-00243-x (PMC10184399; doi:10.1186/s42523-023-00243-x)
Supplement: Supplementary file 8 — Additional file 8. Table S1. The effect of sex and stable isotopes ratio of nitrogen (ẟ15N) on the relative abundance of skin and gut microbiome of adults H. squalostoma [file 42523_2023_243_MOESM8_ESM.docx]

Table S1. The effect of sex and stable isotopes ratio of nitrogen (ẟ^15^N) on the relative abundance of skin and gut microbiome of adults H. squalostoma. These relationships were obtained with a generalized linear model that incorporated only significantly abundant ASVs of adults

|  | **Sources** | **Estimate** | | **Standard error** | **tvalue** | **Pr(>\|t\|)** |
| --- | --- | --- | --- | --- | --- | --- |
| Skin microbiome | Effect on *Staphylococcus sciuri* | | | | | |
|  | intercept | -5.2 | | 78.7 | 25.9 | 0.01 |
|  | ẟ^15^N | 1.9 | | -29.6 | 9.8 | **0.017** |
|  | Sex | -1.9 | | -2.3 | 0.97 | **0.04** |
|  | The effect of sex alone was significant ***p* = 0.007**, deviance = 6.76, residual deviance = 6.1, df = 1 | | | | | |
| Gut microbiome | Effect on unidentified species of *Coprococcus* | | | | | |
|  | intercept | -8.3 | | 11.4 | -0.7 | 0.45 |
|  | ẟ^15^N | 3.4 | | 4.2 | 0.8 | 0.4 |
|  | Sex | -2.1 | | 0.6 | -3.4 | **0.01** |
|  | The effect of sex alone was significant ***p* < 0.001**, deviance = 6, residual deviance = 2.1, df = 1 | | | | | |
|  | Effect on unidentified species of *Clostridium* | | | | | |
|  | intercept | -36.82 | | 34.7 | -1.1 | **0.3** |
|  | ẟ^15^N | -19.3 | | 5129 | -0.004 | **1** |
|  | Sex | 13.7 | | 12.8 | 1.1 | **0.3** |
|  | The effect of sex alone was significant ***p* = 0.02**, deviance = 10.2, residual deviance = 5.6, df = 1 | | | | | |
|  | Effect on unidentified species of *Desulfovibrio* | | | | | |
|  | intercept | -8.4 | 10.2 | | -0.8 | 0.4 |
|  | ẟ^15^N | -0.7 | 0.3 | | -2.1 | 0.08 |
|  | Sex | 3.3 | 3.8 | | 0.9 | 0.4 |
|  | The effect of sex alone was significant ***p* = 0.03**, deviance = 2.4, residual deviance = 51.2, df = 1 | | | | | |
