# Supplementary material for: Parental care contributes to vertical transmission of microbes in a skin-feeding and direct-developing caecilian
Source: Anim Microbiome. 2023 May 15;5:28. doi: 10.1186/s42523-023-00243-x (PMC10184399; doi:10.1186/s42523-023-00243-x)
Supplement: Supplementary file 9 — Additional file 9 Table S2. Tukey post hoc test indicating differences across life stage of skin nitrogen stable isotope (ẟ15N) [file 42523_2023_243_MOESM9_ESM.docx]

Additional file:1 Table S2. Tukey post hoc test indicating differences across life stages of skin nitrogen stable isotope (ẟ^15^N).

| **Sources** | **Estimate** | **Confidence low** | **Confidence high** | ***p*.adjusted** |
| --- | --- | --- | --- | --- |
| Females versus juveniles | 2.56 | 1.83 | 3.28 | **< 0.001** |
| Males versus juveniles | - 3.31 | - 1.63 | - 2.48 | **< 0.001** |
| Females versus males | - 0.752 | - 4.13 | 0.128 | **< 0.1** |
