# Supplementary material for: Parental care contributes to vertical transmission of microbes in a skin-feeding and direct-developing caecilian
Source: Anim Microbiome. 2023 May 15;5:28. doi: 10.1186/s42523-023-00243-x (PMC10184399; doi:10.1186/s42523-023-00243-x)
Supplement: Supplementary file 5 — Additional file 5. Figure S5. Composition and relative abundance of skin bacteria of juveniles and females H. squalostoma according to their reproductive status [file 42523_2023_243_MOESM5_ESM.pdf]

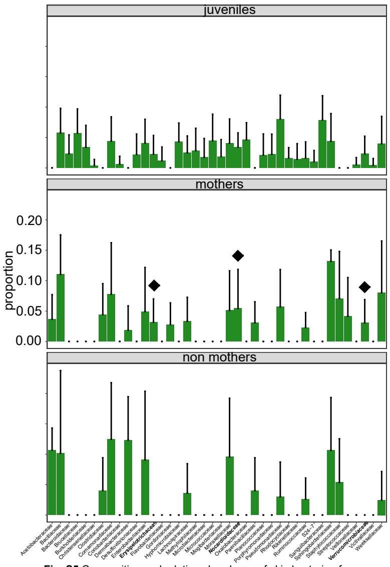

Fig. S5 Composition and relative abundance of skin bacteria of *H. squalostoma* juveniles and females with respect to their reproductive status. Bacteria taxa that were only shared by juveniles (top) and mothers (middle) but not by non-mothers (bottom) are in bold and indicated by the black diamond.
